# Eco-friendly Cu/NiO nanoparticle synthesis: Catalytic potential in isatin-based chalcone synthesis for anticancer activity

**DOI:** 10.1016/j.mex.2023.102471

**Published:** 2023-11-03

**Authors:** Antonius Herry Cahyana, Yosephine Liliana Intan Danar Saputri, Rika Tri Yunarti, Sang Kook Woo

**Affiliations:** a Department of Chemistry, Faculty of Mathematics and Natural Sciences, Universitas Indonesia, Depok 16424, Indonesia; b Department of Chemistry, University of Ulsan, Ulsan 44776, Republic of Korea

**Keywords:** Anticancer, Isatin-based chalcone, Claisen schmidt condensation Cu/NiO nanoparticles, Synthesis of isatin-based chalcone via claisen schmidt condensation

## Abstract

Isatin (1H-indole-2,3‑dione) is a natural heterocyclic compound extracted from various plants and has biological activity as an anticancer agent. Chalcones with the addition of several functional groups (hydroxyl, carboxyl, phenyl, etc.) may become useful templates for the development of new anticancer agents. In this study, we have synthesized Cu/NiO nanoparticles using the sol-gel method involving annona muricata L leaf extract and used as catalysts for the synthesis of isatin-based chalcone. These compounds will be applied as anticancer agents against MCF-7 cancer cell. According to the characterization results using FT-IR, XRD, FESEM-EDS, the crystal size for Cu/NiO nanoparticles was 5.4566 nm and the particle size was 25.081 ± 8.422 nm with irregular spherical shapes. The synthesis of isatin based on chalcone using the reflux method refers to the claisen schmidt condensation reaction using 5% mmol Cu/NiO nanoparticles resulting in yields for each product of 50 % (product 1), 32.37 % (product 2), 24.29 % (product 3), 32.35 % (product 4), 50.86 % (product 5), and 69.88 % (product 6). The effectiveness of the six products against MCF-7 cancer cells can be seen from the IC50 values as follows product 1 (IC50 = 0.00157 µg/ml), product 2 (IC50 = 100.897 µg/ml), product 3 (IC50 = 81.991 µg/ml), product 4 (IC50 = 8107.54 µg/ml), product 5 (IC50 = 77.9291 µg/ml), product 6 (IC50 = 25.4521 µg/ml). Based on the IC50 value obtained, it shows that product 1 and product 6 have strong activity when compared to another product on against MCF-7 cancer cells.

• Acetophenone as a simple ketone was modified to 2-acetylpyridine.
• Modification was performed adding Cu/NiO nanoparticle as catalyst.
• Final products exhibited anticancer activity (MCF-7).

## Graphical abstract

**Figure:**
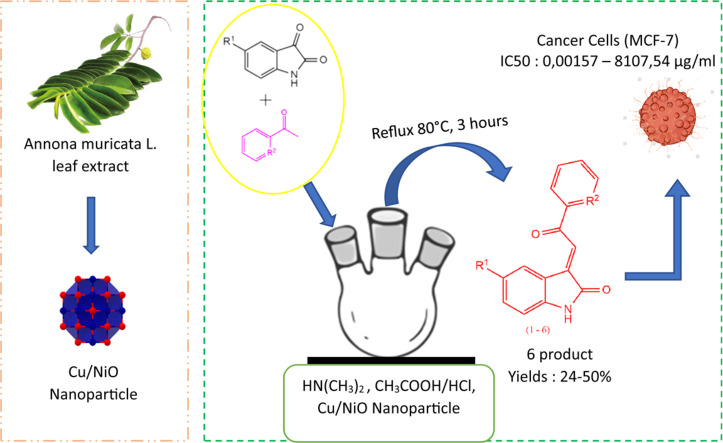
Eco-Friendly Cu/NiO Nanoparticle Synthesis: Catalytic Potential in Isatin-based Chalcone Synthesis for Anticancer Activity

Specifications table

**Table:** 

Subject area:	Chemistry
More specific subject area:	Organic Chemistry
Name of your method:	Synthesis of isatin-based chalcone via claisen schmidt condensation
Name and reference of original method:	NA
Resource availability:	The investigation was conducted in the Laboratory of Organic and Biochemistry, Department of Chemistry, Faculty of Mathematics and Natural Sciences, Universitas Indonesia, Depok. The reagents and chemicals used were purchased from commercial suppliers such as Merck, Sigma-Aldrich. The instruments used were FTIR, UV–Vis and HRMS.

## Introduction

Indonesia is a country rich in biodiversity with a wide variety of plant species. These plants are not only used for ornamental or food purposes but have been traditionally utilized by communities for generations as medicinal remedies [1]. This is due to the presence of naturally occurring chemical compounds in plants, known as primary and secondary metabolites, obtained through metabolic processes. Carbohydrates, proteins, fats, and nucleic acids are categorized as primary metabolites, while alkaloids, steroids, terpenoids, phenolics, saponins, flavonoids, and tannins fall under secondary metabolites [[2], [3]]. Generally, these secondary metabolites can serve as lead compounds in the innovation and development of new drugs with various therapeutic properties, including antioxidants, anticancer agents, antibacterials, antivirals, anti-inflammatories, and inhibitors of carcinogenic effects [3].

Nanotechnology development continues to advance, with researchers in both academic and industrial settings exploring various methods for synthesizing nanoparticles. Nanoparticles can occur naturally or be artificially produced. Nanoparticle synthesis involves creating particles with sizes below 100 nm, exhibiting diverse properties and functions [4]. Several methods can be used for nanoparticle synthesis, including physical, chemical, and biological approaches. In physical and chemical processes, hazardous synthetic reducing agents are often employed. Therefore, strategies have been developed to utilize bioreducing agents derived from natural sources. Biosynthesis of nanoparticles utilizes plant extracts for nanoparticle synthesis [5].

Chalcones are a group of polyphenolic compounds derived from the flavonoid family. Studies have shown that some chalcones may possess therapeutic potential for various diseases. Chalcones, which are aromatic ketones and enones, are known for their anticancer effects [6].

The main structure of chalcone consists of two aromatic rings connected by a three-carbon α,β-unsaturated carbonyl system. Various synthetic compounds containing heterocyclic rings such as pyrazole, indole, etc., have been proven effective as anticancer agents. In addition to their use as anticancer agents against cancer cell lines, heterocyclic analogs have been reported to be effective even against resistant cell lines [7].

Based on this background, research was conducted on the green synthesis of Cu/NiO nanoparticles and their application as catalysts for the synthesis of isatin-based chalcones for anticancer purposes.

## Method details

### Extraction of Annnona muricata L. leaves [8] and phytochemical analysis [9]

Annona muricata L. leaves were collected and thoroughly washed, then air-dried and finely ground using a blender. The Annona muricata L. leaves were macerated with methanol (1: 10). The methanol exract of Annona muricata L. leaves was separated from the residue to obtain the basic extract filtrate. This extract was then partitioned using n-hexane (1: 1) in a separating funnel until complete separation. The basic extract partition was divided into two fractions: the n-hexane fraction (containing non-polar secondary metabolites) and the methanol fraction (containing polar secondary metabolites). Subsequently, the methanol fraction obtained was concentrated using a rotary vacuum evaporator at a temperature of 40 – 50 °C to obtain a dry residue, which was then dissolved in water to obtain the water extract of Annona muricata L. leaves. Finally, phytochemical analysis was conducted on the secondary metabolite compounds like alkaloid, flavonoid, polyphenol, tannin, saponin, terpenoid and steroid. This extract was further used for the synthesis of Cu/NiO nanoparticle.

### Green synthesis of Cu/NiO nanoparticles [10]

Bimetallic nanoparticles were synthesized by dissolving 0.468 gs of Cu(NO_3_)_2_·3H_2_O in 100 mL of distilled water and stirring for 30 min. Then, 14.54 gs of Ni(NO_3_)_2_·6H_2_O were dissolved in 100 mL of distilled water and stirred for 30 min. The Cu(NO_3_)_2_·3H_2_O solution was added to the Ni(NO_3_)_2_·6H_2_O solution and stirred until homogeneous. During the stirring, a 20 mL extract of Annona muricata L. leaves was added. The mixture was then stirred at 70 °C for 12 h. The formed gel was washed with ethanol:distilled water (1:1) and dried. After drying, calcination was performed at 400 °C for 4 h, and the final product was characterized using FT-IR spectroscopy (Shimadzu IRPrestige-21 FTIR in the range of 4000–400 cm^−1^, Europe), XRD (PW3040/60 X'pert PRO PANalytical, Netherlands), FESEM-EDS (JEOL Type JIB 4610F, Japan).

### Synthesis of isatin-based chalcone [11]

Isatin (nitroisatin/5-chloroisatin) 2 mmol and (acetophenone/2-acetylpyridine) 2 mmol were mixed in separate reflux flasks, followed by the addition of two drops of dimethylamine, 2 mL of glacial acetic acid, two drops of concentrated HCl, and 5 % Cu/NiO nanoparticle catalyst. The mixture was then refluxed at 80 °C for 3 h. After refluxing, the formed chalcone was evaporated and washed with distilled water (2 × 5 mL) to remove the acid and dimethylammonium acetate. Component identification was carried out using TLC (Merck TLC Silica Gel 60 F254), FT-IR spectroscopy (Shimadzu IRPrestige-21 FTIR in the range of 4000–400 cm^−1^, Europe), UV–VIS spectroscopy (Shimadzu UV-2450 in the range of 200–800 nm, Japan), and LC-MS (Waters Xevo G2-XS QTof instrument, ACQUITY UPLC®HSS C18 H—Class System, USA), Stuart™ Analog Melting Point Apparatus was used to identify the melting point of the compound

### *In vitro* anticancer assay of isatin-based chalcones (Biofarmaka IPB)

#### Preparation of growth medium

One liter of deionized water is prepared and dissolved with d-MEM or RPMI powder. For d-MEM, 3.7 gs of NaHCO_3_ is added, while for RPMI, 2 gs of NaHCO_3_ is added. The medium is then filtered with a 0.2 µm filter. Finally, FBS and antibiotics are added, and the medium is stored in a refrigerator at 4 °C.

### MTT assay

Cultured viable cells from T25 flasks are subcultured, and 5000 cells/well are cultured in 96-well tissue culture plates and incubated for 24 h in growth media at 37 °C and 5 % CO2. Bioactive compounds are added at various concentrations, 100 µl/well. The control cells do not require treatment. The cells are then incubated for an additional 48 h. 3-(4,5-Dimethylthiazol-2-yl)−2,5-diphenyltetrazolium bromide (MTT) reagent is added and incubated at 37 °C and 5 % CO2 for 4 h. The cell supernatant is removed, and the formazan crystals are dissolved in 70 % ethanol. Optical density (OD) values are obtained using a microplate reader at a wavelength of 565 nm.

## Results and discussion

### Green synthesis of Cu/NiO nanoparticles

The plant used in the synthesis of Cu/NiO nanoparticles in this research is the leaves of Annona muricata L. (soursop leaves). The choice of Annona muricata L. leaves was made because they contain secondary metabolite compounds that have the potential for the synthesis of nano materials.

Phytochemical analysis is a qualitative test conducted to determine the secondary metabolite compounds present in a sample. Below are the results of phytochemical testing of Annona muricata L. leaf extract for the hexane fraction, methanol fraction, and water fraction.

The results of the phytochemical on the water fraction of Annona muricata L. leaf extract (Table 1) showed the presence of alkaloids, flavonoids, saponins, polyphenols, tannins, and terpenoids, consistent with the study conducted by Agu and Okolie in 2017. In this research, the water fraction of the Annona muricata L. leaf extract will be used for the synthesis of Cu/NiO nanoparticles.

**Table 1:** Phytochemical analysis of Annona muricata L. leaf extract.

Secondary Metabolite	Partition			Ref. [12]
	Water Fraction	Metanol Fraction	Hexane Fraction	
Alkaloid	+	–	–	+
Flavonoid	+	–	–	+
Saponin	+	–	–	+
Polyphenols	+	–	–	+
Tannin	+	++	–	+
Terpenoid	+	++	–	+
Steroid	–	++	+	+

*Note:* + (Confirmed).

- (Not confirmed).

Cu/NiO nanoparticles are synthesized using the sol-gel method. In this method, precursors are dissolved in water or alcohol with the assistance of heating and stirring (hydrolysis/alcoholysis), which transforms them into a gel (Bokov et al. 2021). The precursor solution of Cu(NO_3_)_2_·3H_2_O is mixed with the precursor of Ni(NO_3_)_2_·6H_2_O, and then Annona muricata L. leaf extract is added, leading to hydrolysis and condensation processes. Secondary metabolites in the Annona muricata L. leaf extract play a role in the nucleation process, leading to the formation of greenish colloidal or sol Cu/NiO. Subsequently, the colloids formed are subjected to calcination, resulting in the production of pure Cu/NiO nanoparticles.

The synthesized Cu/NiO nanoparticles mediated by Annona muricata L. leaves were successfully synthesized, resulting in spherical materials with a uniform particle size distribution of approximately 25.081 ± 8.422 nm and elemental composition consist of Ni (44.1 %), O (40.2 %), and Cu (7.2 %). The characterization can be seen in Table 2.

**Table 2:** Characterization of Cu/NiO nanoparticles.

Cu/NiO nanoparticle		
IR (cm^−1^)	XRD (nm)	FESEM-EDS (nm)
462 cm^−1^ (Cu/NiO nanoparticle)	5,4566	Spherical,
3464 cm^−1^ (O—H)		25.081 ± 8.422
1034 cm^−1^ (C—H)		Ni (44.1 %),
		O (40.2 %),
		Cu (7.2 %)

### Synthesis of isatin-based chalcone

**Scheme 1:**
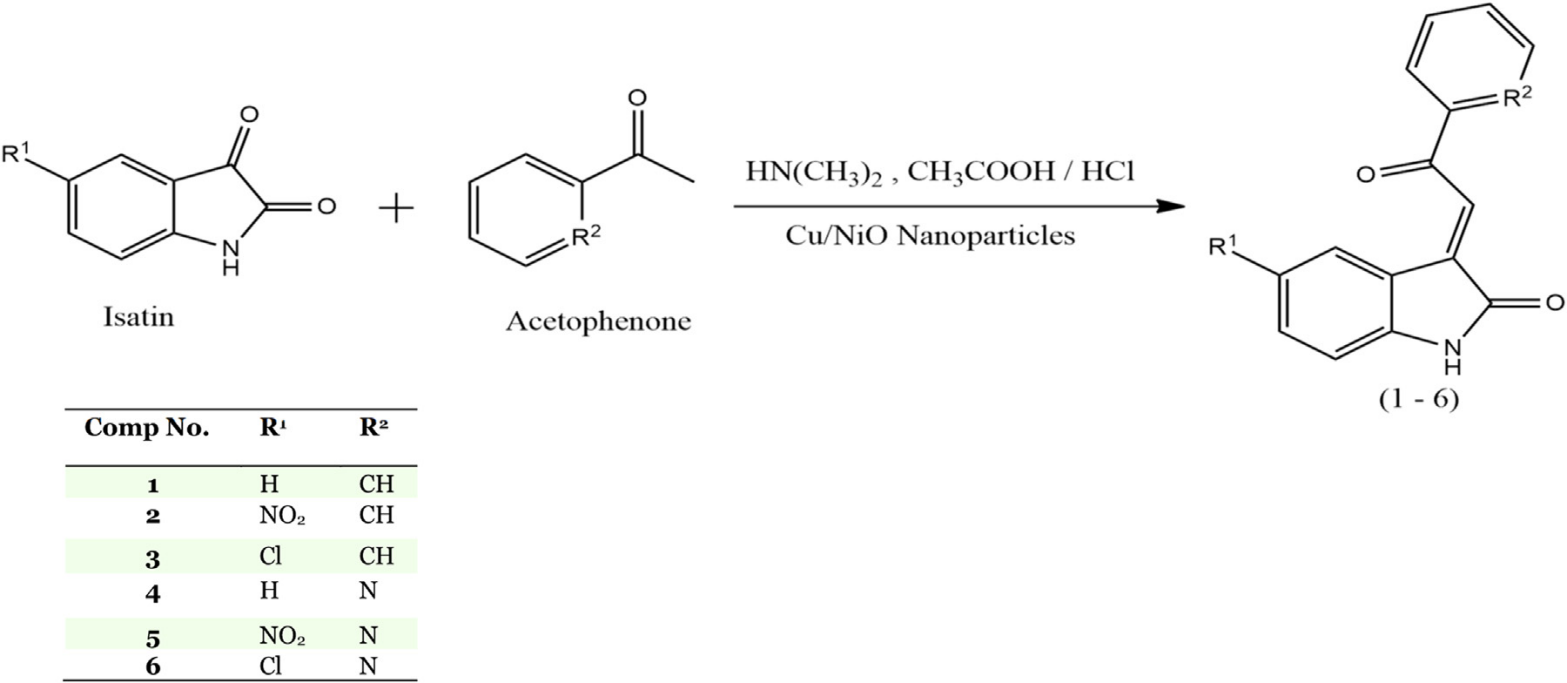
Synthesis reaction of isatin-based chalcone compound.

The isatin-based chalcone compounds, namely compounds 1 - 6 (Scheme 1), were produced using the claisen-schmidt aldol condensation reaction. Isatin, which acts as an electrophile, and acetophenone, which acts as a nucleophile, make up the starting material. Acetophenone possesses a Hα atom in the carbonyl group, allowing it to form a carbanion in a basic environment. Because it may conjugate to form enolate ions, this carbanion is fairly stable. The carbon of the isatin carbonyl group is attacked by the acetophenone carbanion. This nucleophilic addition reaction will result in the formation of β‑hydroxy ketone. The carbonyl group of this β‑hydroxy ketone chemical contains hydrogen, which causes a dehydration reaction to take place when acid is applied, resulting in chalcone compound derivatives. The synthesis of isatin-based chalcone compounds using Cu/NiO nanoparticles was successfully achieved. Then, the synthesized of isatin-based chalcone with Cu/NiO nanoparticle as catalysis were confirmed by Infrared (IR) and High Resolution Mass Spectrometry (HRMS). The findings of HRMS of synthesized compound were found to be very close to the theoretical values. Mass spectra of the synthesized derivatives reflected the characteristic [*M* + *H*]^+^ ion peaks. The yield obtained for each product was 50 % (product 1), 32.37 % (product 2), 24.29 % (product 3), 32.35 % (product 4), 50.86 % (product 5), and 69.88 % (product 6). Complete information about analysis data of the successfully synthesized compounds is shown in Table 3. The effectively generated product was characterized by identifying the functional groups present in the compounds structure utilizing a Fourier Transform InfraRed (FTIR) Spectrometer instrument. The detected wave numbers are listed in Table 4 along with the IR spectra.

**Table 3:** Analysis of synthesis compound (Isatin-based Chalcone).

Product	Molecular formula	Appearance	Melting point	%Yield	MS (*m/z*)	UV–Vis (nm)
1	C_16_H_11_NO_2_	Orange crystalline, solid	184 - 186 °C	50	250.0873	207, 260, 336, and 437
2	C_16_H_10_N_2_O_4_	White crystalline, solid	180 - 182 °C	32.37	295.2632	225 and 336
3	C_16_H_10_N_2_O_4_	Brownish-yellow, solid	201 - 204 °C	24.29	284.0464	212, 255, 335, and 438
4	C_15_H_10_N_2_O_2_	Creamy colored, powder	188 - 192 °C	32.35	251.0826	208 and 254
5	C_15_H_9_N_3_O_4_	Yellow brown, liquid	176 - 180 °C	50.86	295.0593	265, 332 and 384
6	C_15_H_9_ClN_2_O_2_	Dark brownish-black, powder	205 - 211 °C	69.88	285.0427	266 and 413

**Table 4:** Analysis of synthesis compound (Isatin-based Chalcone).

FunctionalGroup	Wavelength number (cm^−1^)					
	Product 1	Product 2	Product 3	Product 4	Product 5	Product 6
N- H stretching vibration	3159	–	–	3180	–	–
C – H stretching vibration	3004	–	3024	–	–	3051
C = O ketone	1710	1748	1720	1707	1748	1707
C = O lactam	1659	–	–	–	–	–
Secondary N – H stretching vibration	1605	1619	–	–	–	–
Aromatic C = C	1455	–	1470	1479	–	1456
Aromatic C – H bending	1225, 756	–	1048	–	–	–
Secondary N —H amide stretching vibration	–	3085	1619	3510, 3397	3330, 1619	1612
Nitro	–	1531, 1340	–	–	1531, 1340	–
Aromatic C – H out of plane	–	845	–	–	831	–
C = C bending vibration	–	743	–	1001, 777	777	–
C - Cl	–	–	777	–	–	784
C – H alkane	–	–	–	2844	–	–
C – N amine	–	–	–	–	1191	–
Aromatic C – H stretching vibration	–	–	–	–	3099	–
Aromatic C – H in plane	–	–	–	–	–	1185
Secondary N – H amine stretching vibration	–	–	–	–	–	2821

### Anti-cancer activity

The MTT assay is a method used to assess cytotoxic activity, specifically the inhibition of cancer cell proliferation. It involves the modification of a tetrazolium salt called MTT to produce a blue formazan product through reduction by cellular enzymes [13]. The IC50 value represents the concentration of a compound that inhibits 50 % of cancer cell proliferation, indicating its potential toxicity [14]. The IC50 value is determined by analyzing the linear regression curve between the percentage of inhibition and the logarithm of the concentration. In the case of isatin-based chalcone compounds tested against MCF-7 cells, the IC50 values can be found in Table 5. According to the National Cancer Institute (NCI), compounds are classified as active when their IC50 values are below 30 µg/ml, moderately active when IC50 values range from 30 µg/ml to 100 µg/ml, and inactive when IC50 values exceed 100 µg/ml [15]. The anticancer activity of the six synthesized isatin-based chalcone products showed varying levels of potency (Table 5), ranging from weak to strong. Products 1 and 6 exhibited strong activity, while products 3 and 5 showed moderate activity. Products 2 and 4 did not exhibit any activity.

**Table 5:** The IC50 values of isatin-based chalcone compounds (products 1 – 6) against MVF-7 breast cancer cells.

Product	Product Name	IC50 (µg/ml)
1	Chalcone	0.00157
2	Chalcone Nitrasi	100.897
3	Chalcone 5-Kloroisatin	81.991
4	Chalcone 2-Acetylpyridine	8107.54
5	Chalcone Nitrasi 2-Acetylpyridine	77.9291
6	Chalcone 5-Kloroisatin 2-Acetylpyridine	25.4521

## Conclusion

Nanoparticles of Cu/NiO mediated by Annona muricata L. leaves were successfully synthesized, resulting in spherical material with a relatively uniform size distribution of approximately 25.081 ± 8.422 nm. The synthesis of isatin-based chalcone compounds using Cu/NiO nanoparticles was successfully performed. Yields for each product were 50 % (product 1), 32.37 % (product 2), 24.29 % (product 3), 32.35 % (product 4), 50.86 % (product 5), and 69.88 % (product 6). The anticancer activity of the six synthesized isatin-based chalcone products ranged from weak to strong. Strong activity was observed in products 1 and 6, while moderate activity was seen in products 3 and 5. Products 2 and 4 showed no activity.

## CRediT authorship contribution statement

**Antonius Herry Cahyana:** Investigation, Validation, Data curation, Writing – original draft. **Yosephine Liliana Intan Danar Saputri:** Methodology, Supervision, Conceptualization, Writing – review & editing. **Rika Tri Yunarti:** Methodology, Supervision. **Sang Kook Woo:** Supervision.

## Declaration of Competing Interest

The authors declare that they have no known competing financial interests or personal relationships that could have appeared to influence the work reported in this paper.

## Data Availability

The authors do not have permission to share data.
